# Natural sustainable and eco-friendly catalyst utilizing lupine peel waste for efficient degradation of organic pollutants via Fenton-like reactions

**DOI:** 10.1038/s41598-025-08771-z

**Published:** 2025-07-03

**Authors:** Nour W. Sabry, Ibrahim Naeem, Seed A. Hassanien, Osama Abuzalat, Ahmad Baraka

**Affiliations:** Department of Chemical Engineering, Military Technical College, Cairo, Egypt

**Keywords:** Eco-friendly catalyst, Fenton-like process, Methylene blue, Decomposition, Chemistry, Catalysis, Catalyst synthesis

## Abstract

Peels of agricultural wastes are gaining new outlook recognition as affordable and effective catalysts for organic pollutants. This study investigates the potential of untreated (S1) and alkaline-treated Lupine Peels Powder (S2) as an eco-friendly biomaterial for degrading cationic methylene blue (MB) from wastewater. The findings pertain to the catalytic degradation of MB and the improved degradation observed following alkaline treatment. The presence of active manganese sites on the surface of S2 samples enhances the catalytic degradation of MB; as manganese sites accelerate H_2_O_2_ breakdown, forming ˙OH radicals that degrade MB. Optimizing H_2_O_2_ concentration, MB concentration, and temperature is crucial for high efficiency. The optimum removal efficiency reaches (82.37%) occurs at 25 °C with 6% of H_2_O_2_ for MB with *C*_o_= 15 ppm which is very close to 4% of H_2_O_2_ so, the optimum concentration of H_2_O_2_ is 4% for *C*_o_ ranges from 5 to 15 ppm. Higher MB concentrations and H_2_O_2_ levels improve efficiency to a specific limit, but higher temperatures reduce it by accelerating H_2_O_2_ decomposition. XPS characterization analysis reveals that NaOH treatment increases the visibility of metal elements such as manganese, copper, and iron, providing better insights into the surface composition and its catalytic potential for MB degradation. In general, NaOH treatment enhances MB removal by improving the surface activity of lupine peels, likely through structural and compositional modifications.

## Introduction

One of the major environmental issues facing the world is pollution, including the contamination of water by organic dyes^1,2^. Dyes are harmful organic pollutants that originate from industrial waste in sectors such as textiles, leather, food, cosmetics, and rubber, and they present a significant risk to environmental health and safety^3^. The global textile industry consumes over 10^7^ kg of dye annually, with an estimated 90% of this dye being used on fabrics. As a result, around 10% or more of these dyes are released into wastewater without treatment by the textile industry worldwide^4^.

Various physical, biological, and chemical techniques have been employed to treat wastewater containing dyes^5^. Most of these methods have drawbacks, including the production of hazardous sludge, high costs for operation and maintenance, and the complexity of the processes involved in treatment^6^. It is essential to explore innovative methods for efficiently treating dye-contaminated wastewater. Advanced oxidation processes (AOPs), such as catalytic oxidation^7^, the Fenton process^8^, the photo-Fenton process, photocatalysis, and bio-catalysis have gained significant attention in recent years for their effectiveness in addressing this issue^9^.

In recent years, various types of photocatalysts have emerged such as Titanium dioxide (TiO_2_), which has become a highly effective photocatalyst for environmental^10^. Another photocatalyst is the yttrium-perylenetetracarboxylate (Y-PTC) metal-organic framework (MOF) evaluates the photocatalytic activity of degrading methylene blue and methyl orange when exposed to visible light^11^. A recent development in tackling dye-contaminated water is the use of Fenton and Fenton-like reactions. These processes decompose H_2_O_2_ to produce powerful oxidative species known as hydroxyl radicals (^**·**^OH), which effectively break down organic pollutants such as methylene blue, methyl orange, and even commercial dyes^12^. As well as H_2_O_2_, is effective in treating various industrial wastes including nitrites, hypochlorites, chlorides, cyanides, thiocyanates, and organic matter^13^.

A novel cobalt-doped carbon aerogel (Co-CA) demonstrates high efficiency in degrading methylene blue (MB) through a heterogeneous Fenton-like reaction when H_2_O_2_ and carbonate ions are present. It was discovered that Fe_3_O_4_ nanoparticles can serve as an effective catalyst for the degradation of methylene blue dye in aqueous solution within a Fenton-like system^14^. Although this technology offers benefits, it produces by-products after dye degradation, which complicates the practical use of the homogeneous Fenton reaction^15^.

The Fenton process (homogeneous catalysis) has been successfully applied to eliminate persistent organic pollutants^16^, but it faces challenges such as pH sensitivity and the generation of ferric hydroxide sludge which can result in pollution and higher costs^17^. To mitigate these limitations, a range of heterogeneous catalysts, including iron-pillared clays^18^, iron-containing zeolites^19^, and iron minerals^20^, have been employed in Fenton reactions. Research has shown that heterogeneous Fenton reactions can address the shortcomings of homogeneous catalysis effectively^21,22^.

Heterogeneous Fenton-like catalysis provides several benefits, including environmental sustainability, energy efficiency^23^, recyclability^24^, and broad applicability in breaking down a wide range of persistent organic pollutants, such as dyes^25^, fertilizers^26^, benzene and its derivatives^27^, and pharmaceuticals^28^.

Biological treatment methods are regarded as the most effective approach for treating wastewater^29^. Enzymatic wastewater treatment surpasses microbial methods due to its shorter treatment duration, lack of a lag phase, capability to decolorize effluents with higher dye concentrations, ease of process management, and reduced sludge volume^30^.

Recently, agricultural wastes have been utilized as a novel Fenton-like catalyst. A research effort has explored the use of peanut shell ash (PSA) as a novel Fenton-like catalyst, impregnated with iron (Fe^3+^), to investigate the decolorization and degradation of MB. The decolorization efficiency reached 84% using 0.2 g of catalyst, and 16 mM of hydrogen peroxide, at a temperature of 30 °C, a pH of 3, and an initial MB concentration of 15 mg/l. Thus, the study concluded that the peanut shell ash-based Fenton-like catalyst exhibits effective decolorization and degradation efficiency within acidic pH ranges (pH = 3–5)^31^.

Potato peels and soybean hulls are types of agro-industrial waste that provide a rich source of effective enzymes, such as peroxidases, useful for oxidation reactions for degradation of anthraquinone dye Acid Violet 109. When optimal conditions are met, potato peel peroxidase and soybean hull peroxidase achieved biodegradation rates of 72.78% and 66.12%, respectively^32^.

The decolorization of Acid Red 1 (AR1) in aqueous solution was studied using a Fenton-like process. Various reaction parameters, including different iron ion loadings on rice husk ash (RHA), were examined. Under optimal conditions, specifically, a catalyst dosage of 5.0 g.l⁻^1^, an initial pH of 2.0, with H_2_O_2_ concentration at 8 mM, and an initial AR1 concentration of 50 mg.l⁻^1^ at 30 °C; a 96% decolorization efficiency of AR1 was achieved within 120 min^33^.

Lupine peels, a sustainably sourced and cost-effective agricultural waste, were selected for catalytic experiments and recognized as a promising candidate for practical catalysis applications. This study focuses on evaluating the degradation efficiency of methylene blue (MB) using an eco-friendly lupine peel-based catalyst, utilizing active sites in the natural composition of lupine peels to drive Fenton-like reactions for enhanced catalytic performance. These reactions facilitate the decomposition of hydrogen peroxide, generating reactive oxygen species (ROS) responsible for breaking down cationic MB⁺^12,34,35^.

To investigate the structural and chemical properties, lupine peels were characterized using SEM/EDX and XPS. These techniques were employed to analyze the elemental composition of both untreated and alkali-treated samples and to assess oxidation states before and after catalytic activity. XPS, widely used for surface analysis, plays a crucial role in various fields, including corrosion studies, catalysis, electronics, nanomaterials, biomedicine, mineral processing, and the automotive and aerospace industries^35^.

Several factors affecting the degradation efficiency of methylene blue (MB) were analyzed to determine optimal conditions for the design framework, including the initial concentrations of H_2_O_2_ and MB, as well as temperature variations. These parameters must be meticulously controlled and integrated into industrial design considerations. Additionally, an alkaline treatment was applied to lupine peels following H_2_O_2_ treatment to evaluate its impact on their catalytic activity.

## Experimental

### Lupine peel catalyst preparation and characterization

Egyptian yellow lupine was obtained from a local market in Egypt. The lupine was soaked in DI water for 24 h, after which the peels were removed and collected. The peels were then thoroughly rinsed with DI water three times and left for three days in the air at ambient conditions to ensure complete dryness. After drying, the peels were ground and sieved to obtain a powder with a mesh size of 0.5 mm. The powder was divided into two portions: one remained untreated (**S1**), while the other was treated with a NaOH solution (**S2**). For preparing **S2**, 0.25 g of lupine peel powder was immersed in a deionized water solution alkalized with 5% NaOH. The mixture was stirred using a shaker at 150 rpm for 2 h at room temperature. After treatment, **S2** was filtered to separate it from the solution and rinsed several times with DI water until the wash reached a neutral pH.

SEM/EDX (Thermo Scientific^™^ Phenom^™^) analysis investigates both the surface morphology and elemental composition of the sample, highlighting differences between (S1) and (S2) samples. XPS (X-ray photoelectron spectroscopy) was conducted using an (Escalab-210 electron spectrophotometer (Spain)) which is an analytical method used to examine the surface chemistry of the material, offering detailed insights into the elemental composition at depths of 1–10 nm with a sensitivity of 0.1-1 atomic percent for most elements, making it a crucial tool for studying surface properties. The chemical state and electronic structure of the elements located within the top few nanometers of the material’s surface were also investigated. Moreover, it provides information about the oxidation state of the elements that existed.

Determining the pH_pzc_ of the catalyst is important for understanding the organic-pollutant degradation mechanism based on H_2_O_2_ decomposition. The drift method was applied for such a purpose^36^. To determine the pH_pzc_ of S1 and S2, 100 mL of 0.01 N NaCl solution was added to a series of flasks. The pH of each solution was adjusted to values between 2.0 and 12.0 using standard HCl and NaOH solutions. The initial pH of each solution was measured with a calibrated pH meter and recorded as pH _initial_. Subsequently, 0.1 g of either raw or treated lupine peels (YLPP or T-YLPP) was added to each flask. After 24 hours of contact, the final pH values (pH_final_) were recorded. The difference between final and initial pH (pH_final_ − pH_initial_) was calculated and plotted against pH_initial_ to determine the pH_pzc_ of the lupine peel samples. The pH_PZC_ values of S1 and S2 were determined to be 5.2 and 4.4 respectively. The initial pH value of MB-solutions was 5.9 which is higher than the pH_PZC_ of both S1 and S2, a condition which promotes suitable attraction of MB^+^ towards S1 and S2 particles’-surface and also promotes the generation of reactive oxidative species in processes such as Fenton-like reactions, thereby enhancing the degradation of MB^+^.

### Methylene blue degradation by lupine peel catalyst

Catalytic degradation experiments for MB were carried out in a 50 ml beaker. From a 1000 ml stock solution of 20 ppm MB, the required volume was transferred to the beaker to prepare MB concentrations of 5, 10, and 15 ppm, with 0.25 g of lupine catalyst added. A 50% hydrogen peroxide solution was used to prepare H_2_O_2_ solutions of varying concentrations (0%, 2%, 4%, 6%, 8%, and 10%). The beaker was then placed in an incubator shaker, with experiments conducted at an agitation speed of 150 rpm. The temperature was applied at 25 °C, 35 °C, and 45 °C for all experiments.

The efficiency of MB dye removal is represented as the percentage ratio of the absorbance of the decolorized dye to the absorbance of the original dye as in Eq. (1)^37,38^.

1 $${\text{Removal efficiency }}\% {\text{ }} = C_{{\text{o}}} -C_{{\text{t}}} /C_{{\text{o}}} \times {\text{1}}00$$

Where *C*_o_ and *C*_t_ (ppm) is the initial concentration and concentration at a certain time *t* respectively.

## Results and discussion

### Characterization

**Fig. 1:**
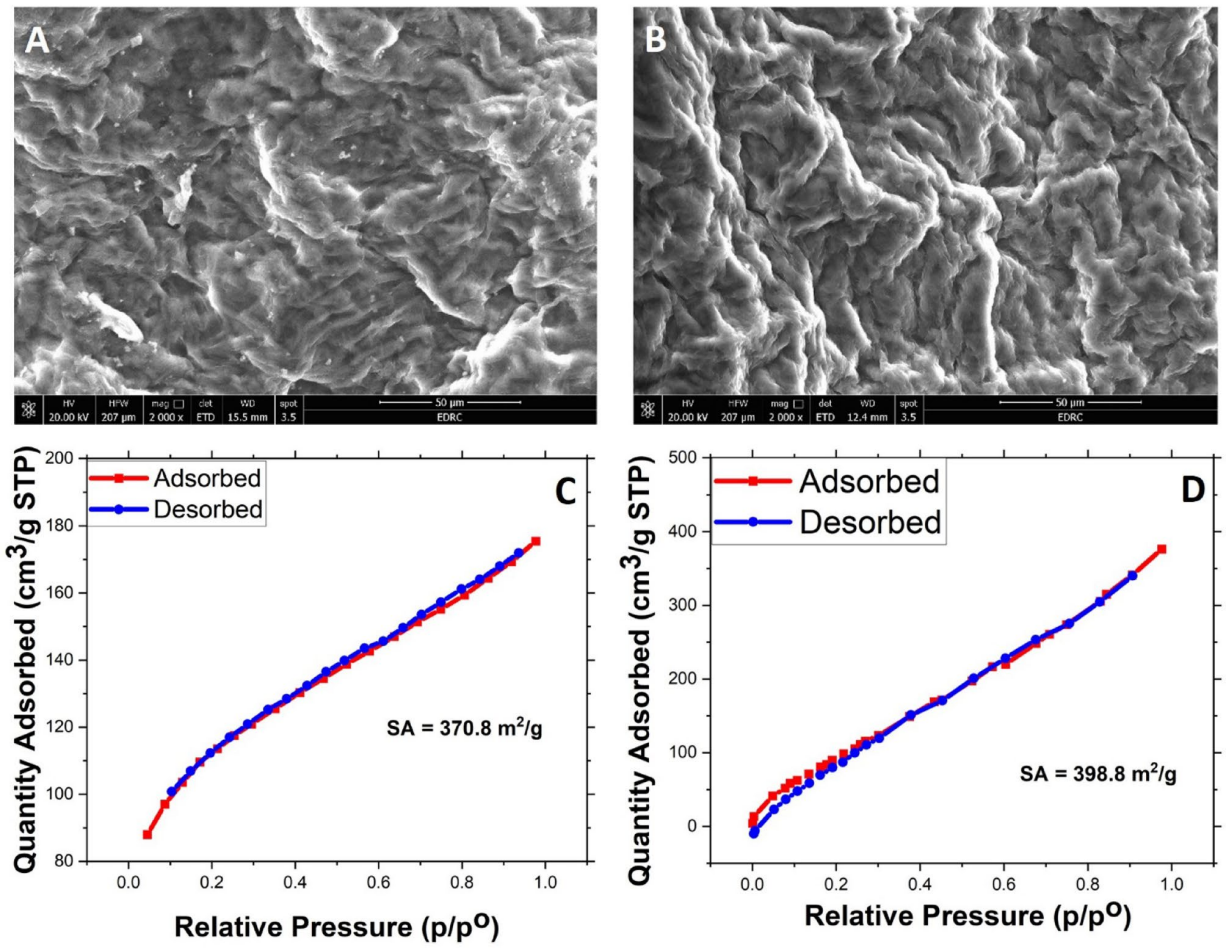
SEM images of (**A**) S1, (**B**) S2, and N_2_ adsorption –desorption isotherm at 77 K of (**C**) S1, and (**D**) S2.

Figure 1 shows the SEM surface images for S1 (a) and S2 (b). Both surfaces can be termed rough random-corrugated, however, the S2 surface is rougher which comes from alkaline treatment where some deprivation of cellulose, hemicellulose and lignin should occur. Such deprivation partially chemically-eroded these natural polymers^39–41^. BET analysis was performed for the samples and the single-point method gave surface area of 370.8 and 398.8 for S1 and S2 respectively as shown in Fig. 1 (C, D). Such an increase in the surface area of S2 concerning that of S1 should come from the mentioned material deprivation. Also, the S2 image reveals a clean surface, compared to the S1 image, with no debris accumulation. Importantly, despite the deprivation, S2 texture stays preserved after.

the alkaline treatment process.

EDX analysis was conducted for both S1 and S2, as presented in Table 1. First, the given percentage sets of S1 and S2 are similar to a great extent which reflects the retained integrity of lupin peel texture after alkaline treatment. Second, the observed slight changes in some percentages give some semantics. The decrease of nitrogen content from 13.1 to 11% should be attributed to some loss of proteins and/or other nitrogenous compound present in texture, especially proteins which are sensitive to alkaline hydrolysis^42^. The increase of oxygen content from 44.1 to 46.9% could be related to surface-exposure of cellulose/lignin parts of lupin peels^41^.

Moreover, other metals such as magnesium, calcium, and manganese (Mn) were detected with small percentages. Notably, the atomic percentages of magnesium, calcium, and manganese are significantly higher in S2 compared to S1. In particular, manganese, a transition metal capable of existing in multiple oxidation states, may play a crucial role in Fenton-like reactions. The catalytic degradation via the Fenton-like reaction is more prominent in S2, as the atomic percentage of manganese increased from 0.1 to 0.2%; this suggests a rise in active sites, enhancing H_2_O_2_ decomposition and generating reactive oxygen species, which promotes the breakdown of organic pollutants such as MB.

**Table 1:** Elemental composition of S1 and S2 based on EDX analysis.

Atom %	C	*N*	O	Mg	S	K	Ca	Mn	Fe	Cu
S1	41.8	13.1	44.1	0.2	0.1	0.5	0.1	0.1	-	-
S2	41.1	11.0	46.9	0.4	0.1	0.0	0.2	0.2	-	-

**Fig. 2:**
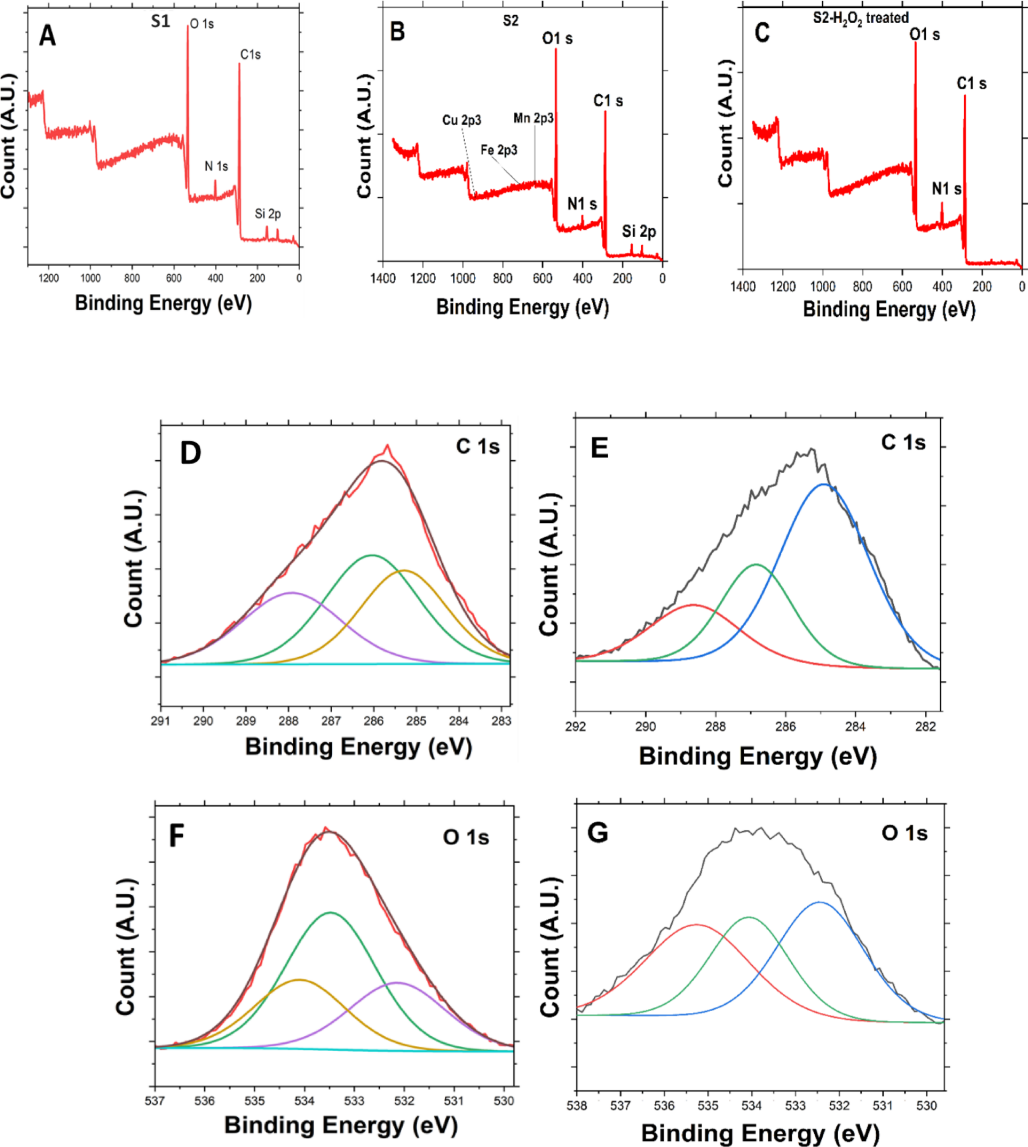

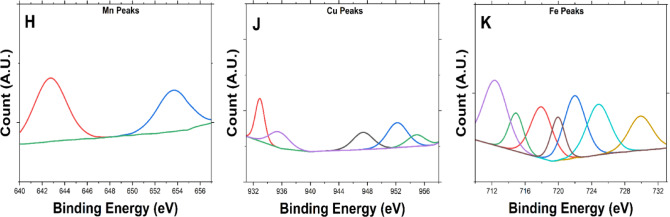
XPS Binding Energy Curves for (**A**) **S1**, (**B**, **D**, **F**) **S2** for T-YLPP, and (**C**, **E**, **G**) for **S2-**H_2_O_2_ treated; (**H**, **J**, **K**) for Mn, Cu, and Fe Peaks.

For further understanding of the S1 and S2 samples, XPS was utilized to investigate the elemental states of both samples before H_2_O_2_ decomposition. This analysis highlights changes in elemental composition resulting from alkali treatment. As shown in Fig. 2, multiple peaks emerge at various binding energies, with significant differences observed between Fig. 2 (A) and (B). In the S2 sample depicted in Fig. 2 (B), manganese, copper, and iron atoms become detectable through XPS analysis. This suggests that the chemical treatment unveils these metals, which were previously obscured by organic material and impurities. The alkali treatment effectively removes these masking layers, enriching specific elements or oxidation states on the surface and thereby improving their detectability via XPS.

Metals in their ionic form, whether dissolved in solution or weakly bound to organic compounds, typically generate weak or indistinct XPS signals due to their unfavorable binding energies for detection. However, chemical modification transforms these ions into metal hydroxides, altering their chemical environment. This conversion sharpens their XPS peaks, making them more distinct. Additionally, metal hydroxides precipitate as solids on surfaces, increasing their surface concentration and enhancing detection by the surface-sensitive XPS technique, which analyzes only the top few nanometers of a sample.

The notable differences between S1 and S2 are observed with an increase in oxygen percentage from 25 to 26.08%, suggesting a higher presence of oxygen-containing functional groups like hydroxyl (–OH), carbonyl (C = O), and carboxyl (–COOH) in S2.

In S2, the elements are present in varying proportions, with the majority being carbon (65.08%), followed by oxygen (26.08%), while the metals are detected in trace amounts (Mn: 0.26%, Cu: 0.22%, Fe: 0.11%). O 1s (~ 534.14 eV), the high binding energy suggests oxygen associated with hydroxyl or carboxylate groups, indicating chemical modification or surface functionalization^43^. C 1s (286.99 eV) is indicative of oxidized carbon species, such as C = O or C-OH, often resulting from chemical treatments^44^. N 1s (401.59 eV) suggests its presence as an oxidized nitrogen species (e.g., amides or nitrates)^45^. Si 2p (103.81 eV), the binding energy is consistent with silica (SiO_2_)^46^.

Transition Metals such as Cu 2p3 (933.38 eV) correspond to Cu^2+^, likely present as CuO or Cu(OH)_2_ after treatment^47^, additionally peaks at various scans were investigated as shown in Fig. 2 (J). Fe 2p3 (712.08 eV), this narrow binding energy suggests the existence of Fe^3+^, potentially in an oxidized form like Fe_2_O_3_ or FeOOH^48,49^; furthermore, peaks observed in different scans were analyzed, as illustrated in Fig. 2 (K). Mn 2p3 (645.19 eV) suggests Mn^3+^ or Mn^4+^ species, indicative of manganese oxides or hydroxides formed during NaOH treatment. Mn^4+^ is highly suggested as the binding energy (642.72 eV)^50,51^ as shown in Fig. 2 (H) at the first scan falls within the high range and high atomic percentage which is typically associated with manganese in the Mn^4+^ oxidation state as illustrated in Table 2, which is found in compounds such as manganese dioxide (MnO_2_)^52^.

A third sample, S2-H_2_O_2_ was investigated aimed to observe changes in oxidation states in S2 following H_2_O_2_ treatment. Figure 2 (C) presents the XPS survey spectra of S2 after H_2_O_2_ treatment with 4% H_2_O_2_ concentration, highlighting notable differences between before and after H_2_O_2_ treatment as depicted in Fig. 2 (D) and Fig. 2 (E) respectively. The increase in C 1s Scan A from (287.92 to 288.62 eV) suggests stronger oxidation, likely due to the formation of more carbon-oxygen functional groups (such as carboxyl or carbonate species)^53^. The decrease in C 1s Scan B suggests a reduction in lower oxidation states or removal of some hydrocarbon-like carbon. The binding energy decreased from (285.29 eV to 284.8 eV). A decrease in BE generally means less oxidation (or a shift toward a more reduced carbon state)^54^.

Carbon in C–C (sp^2^ or sp^3^ bonds, hydrocarbon-like carbon) typically appears around 284.5–285.0 eV, while oxidized carbon (C–O, C = O, O–C = O) appears at higher BE (> 285.5 eV). This suggests that some oxidized carbon species were removed or reduced, leaving more sp^2^/sp^3^ carbon. Indicates a reduction in oxidation states (less oxygen-functionalized carbon). Suggests the removal of some surface contaminants, revealing more hydrocarbon-like (sp^2^/sp^3^) carbon. Likely shows an increase in graphitic carbon exposure after the H_2_O_2_ reaction^54^.

Changes in Carbon (C 1s) peak BE shift (286.99 eV increased to 287.39 eV after H_2_O_2_ treatment) suggest the formation of oxidized carbon species (C = O, COO^**–**^). The overall percentage of carbon increased from 65.08 to 67.84% after H_2_O_2_ treatment. Moreover, an increase in FWHM (4.45 to 5.3 eV) indicates a greater variety of carbon oxidation states after H_2_O_2_ treatment^55^.

According to O 1s, after H_2_O_2_ treatment, the binding energies in the sample increase, indicating a greater overall oxidation state. The atomic percentage of oxygen also rises due to the oxidizing effect of H_2_O_2_, which introduces oxygen-containing functional groups on the surface. This additional oxygen incorporation leads to higher binding energies, while broader peaks suggest an increased oxygen content^56^.

The total O 1s peak for S2 shifted from 533.56 eV without H_2_O_2_ treatment to 535.24 eV (S2-H_2_O_2_ treated). Higher BE shift suggests an increase in oxidation due to new oxygen species, possibly hydroxyl (–OH) or peroxo (–O–O–) groups^57^. O 1s Scan A (C = O, C–OH) increased from 532.13 Ev in Fig. 2 (F) to 532.44 eV as shown in Fig. 2 (G) which indicates a growth in oxygen-containing functional groups like carbonyl (C = O) and hydroxyl (C–OH) after oxidation. O 1s Scan B (Carboxyl group) moved from 533.9 eV to 534.06 eV suggesting slight restructuring, possibly removing weakly bound oxygen species and introducing more stable oxidation states^58^.

The Oxygen (O 1s) peak BE shift (534.14 eV to 534.75 eV) increased which suggests increased oxidation due to H_2_O_2_ treatment. Higher Area (182384.16 to 150988.26) and Atomic % (27.39–26.08%) confirms more oxygen-containing species after H_2_O_2_ exposure^59^.

A decrease in (N 1s) with lower Area (13557.22 to 21096.71) and Atomic% (3.52–4.77%) before H_2_O_2_ means that H_2_O_2_ increased nitrogen content, possibly by incorporating nitrogen into oxygenated groups^60^.

There are some reasons for not detecting minerals like manganese, iron, and copper by XPS in the S2-H_2_O_2_ sample; the first is oxidation and dissolution where H_2_O_2_ is a strong oxidizing agent that can react with specific minerals, altering their composition or even dissolving them, especially those containing iron, manganese, or other redox-sensitive elements^61^. Surface modification where the introduction of oxygen functional groups can change the surface chemistry, potentially masking the signals of some minerals in XPS. Structural alterations where H_2_O_2_ may break down or restructure some minerals, leading to phase transformations or amorphization, making them undetectable by conventional methods^62^. Element Leaching where some minerals might release ions into the solution upon reaction with H_2_O_2_, effectively reducing their detectable presence in the solid sample^63^.

**Table 2:** Outputs of binding energies and atomic% by XPS of S1, S2, and S2-H_2_O_2_ treated peels.

Name	Peak BE	FWHM eV	Area (P) CPS.eV	Atomic %
**S1**				
O 1s	534.31	4.96	115139.8	25
C 1s	287.16	5.37	118956.4	65.88
N 1s	401.88	4.02	11550.65	3.77
Si 2p	103.71	5.27	8588.75	5.35
**S2**				
O 1s	534.14	3.61	150988.3	26.08
C 1s	286.99	4.45	147683.1	65.08
N 1s	401.59	4.25	13557.22	3.52
Si 2p	103.81	3.61	9565	4.74
Cu 2p3	933.38	1.18	5153.09	0.22
Fe 2p3	712.08	0.47	2112.69	0.11
Mn 2p3	645.19	0.85	4305.01	0.26
**Mn peaks**				
Mn 2p	642.72	3.37	390.46	60.39
Mn 2p Scan A	653.64	3.37	254.16	39.61
**Cu peaks**				
Cu2p	941.36	3.37	187.53	18.09
Cu2p Scan A	947.39	3.37	175.45	17.03
Cu2p Scan B	932.92	1.48	191.61	18.31
Cu2p Scan C	952.17	3.37	255.74	24.96
Cu2p Scan D	954.41	0.82	55.45	5.43
Cu2p Scan E	936.55	3.37	168.7	16.18
**Fe peaks**				
Fe2p	720.13	0.55	51.18	5.77
Fe2p Scan A	721.85	0.5	45.08	5.09
Fe2p Scan B	716.27	3.37	227.14	25.55
Fe2p Scan C	712.73	3.37	178.27	20
Fe2p Scan D	729.88	3.37	140.73	16
Fe2p Scan E	724.81	3.37	207.92	23.54
Fe2p Scan F	721.26	0.48	35.84	4.05
**S2-H**_**2**_**O**_**2**_ **treated**				
N 1s	402.11	4.41	21096.71	4.77
O 1s	534.75	4.85	182384.2	27.39
C 1s	287.39	5.3	177081.3	67.84

### Catalytic effect versus adsorptive performance for S1 and S2

Before analyzing the impact of various parameters in MB degradation, it is essential to determine whether MB removal occurs through catalysis, simple adsorption, or a combination of both. To assess this, removal efficiencies were compared under three different conditions: catalytic action (adsorbent + H_2_O_2_), adsorption alone (adsorbent), and H_2_O_2_ alone. The experimental conditions for evaluating catalytic and adsorption reactions in S1 were selected as follows: The maximum H_2_O_2_ concentration was set at 10% to assess its impact; moreover, an intermediate concentration of 4% was chosen for an initial MB concentration of 10 ppm at 25 °C to compare the differences.

**Fig. 3:**
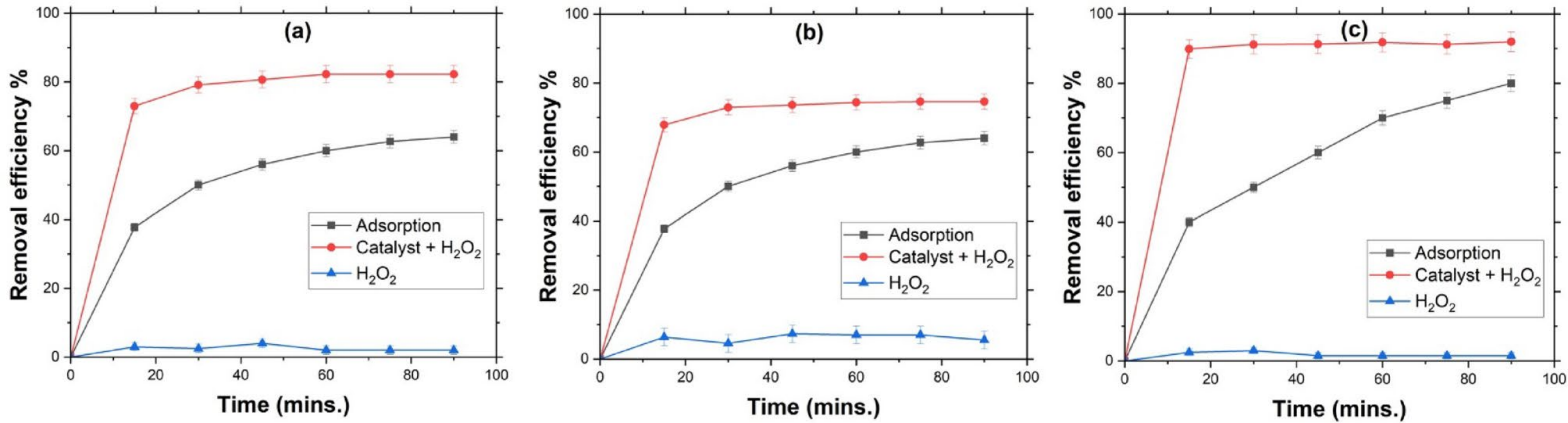
Comparison between the performance of lupine peels as catalyst and adsorbent for MB degradation (*C*_o_ = 10 ppm, *T* = 25 ºC); (**a**) H_2_O_2_ = 4%, (**b**) H_2_O_2_ = 10% for **S1**, and (**c**) H_2_O_2_ = 4% for **S2**.

As depicted in Fig. 3, the lupine peel catalyst, when combined with hydrogen peroxide at concentrations of 4% and 10% exhibited significantly higher removal efficiency than adsorption alone. H_2_O_2_ by itself exhibits the lowest removal efficiency for MB due to its relatively low oxidation potential compared to radical species^31^. These results strongly suggest that the active sites on lupine peels play a crucial role in catalyzing MB degradation, thereby enhancing its removal. The findings highlight the catalyst’s essential role in MB elimination, likely due to the presence of active Mn sites in lupine peels, as confirmed by EDX analysis for both S1 and S2 samples. These Mn sites facilitate H_2_O_2_ decomposition, boosting the generation of ˙OH radicals responsible for breaking down MB contaminants. Increasing the H_2_O_2_ concentration from 4 to 10% leads to a lower MB removal efficiency over 90 min compared to a 4% concentration as shown in Fig. 3 (b). The maximum removal efficiency at 10% H_2_O_2_ is 74.58%, whereas at 4%, it reaches 82.27% as depicted in Fig. 3 (a). This suggests that a higher concentration is not ideal for a 10 ppm initial concentration at 25 °C. From the study of the catalytic effect, it was concluded that the variation in H_2_O_2_ concentration plays a crucial role in MB removal and degradation enhancement. Therefore, it should be carefully considered and further examined.

A similar approach was applied to S2 and a similar trend was observed in S1, as the catalytic effect demonstrated the highest removal efficiency compared to adsorption or the effect of H_2_O_2_ alone. H_2_O_2_ alone resulted in the lowest removal percentage, with no observable removal occurring. When using a lupine peel catalyst with 4% H_2_O_2_, a 92% removal efficiency was achieved, as shown in Fig. 3 (c), whereas adsorption alone led to only 80% removal. Comparing S2 to S1, it was found that at a 4% H_2_O_2_ concentration, 25 °C, and 10 ppm of MB, S2 exhibited the highest removal efficiency, increasing from 82 to 92%. These findings support the catalytic effect after alkaline treatment, highlighting an overall enhancement in MB removal efficiency.

### MB degradation by S1

#### Effect of H_2_O_2_ concentration

**Fig. 4:**
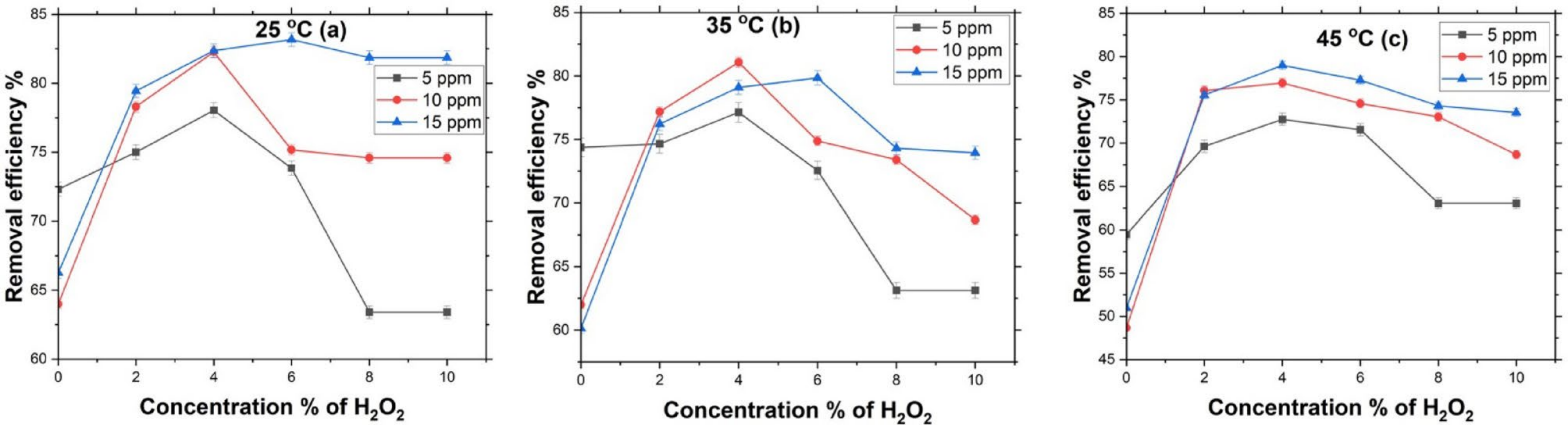
Effect of initial different concentrations of H_2_O_2_ at (0%, 2%, 4%, 6%, 8%, and 10%) and its effect on the initial concentration of MB. (*C*_o_ = 5, 10, and 15 ppm at (**a**) 25 ^o^C, (**b**) 35 ^o^C, and (**c**) 45 ^o^C).

A key factor in this process is the hydrogen peroxide concentration, as it directly influences the production of hydroxyl radicals. To examine its impact on MB degradation efficiency, experiments were carried out using H_2_O_2_ concentrations ranging from 0 to 10%, with lupine peels (0.25 g) at 150 rpm and 25 °C, and initial MB concentrations (*C*_o_) of 5, 10, and 15 ppm. The results, presented in Fig. 4 (a), show that for 5 ppm MB at 25 °C, the optimal H_2_O_2_ concentration is 4%, yielding the highest removal efficiency. As the H_2_O_2_ concentration increased from 2 to 4%, the removal efficiency improved from 75 to 78.06%. However, beyond 4%, the efficiency declined, decreasing to 73.84% at 6% H_2_O_2_ concentration and further dropping to 63.4% at 8%. At 10% H_2_O_2_ concentration, the removal efficiency plateaued, showing no significant difference from the 8% concentration result.

This decline in MB removal efficiency at higher H_2_O_2_ concentrations is likely due to the formation of hydroperoxyl radicals, which are less reactive than hydroxyl radicals. Additionally, excessive H_2_O_2_ can lead to radical recombination, reducing the availability of hydroxyl radicals for dye degradation. The equations described in Eq. (2) to (4) illustrate the formation of hydroperoxyl radicals at elevated H_2_O_2_ concentrations, which contribute to the reduced efficiency of dye removal^24,64,65^.

2 $${^\cdot\text{OH}} + {^\cdot\text{OH}} \to {\text{H}}_{{\text{2}}} {\text{O}}_{{\text{2}}}$$

3 $${^\cdot\text{OH}} + {\text{HO}^\cdot}_{{{\text{2 }} \to }} {\text{O}}_{{\text{2}}} + {\text{ H}}_{{\text{2}}} {\text{O}}$$

4 $${\text{H}}_{{\text{2}}} {\text{O}}_{{\text{2}}} + {^\cdot\text{OH}} \to {\text{HO}^\cdot}_{{\text{2}}} + {\text{H}}_{{\text{2}}} {\text{O}}$$

For 5 ppm in Fig. 4 (a), the MB removal percentage was 72.3% before the addition of H_2_O_2_, mainly due to adsorption onto the lupine peels. However, H_2_O_2_ concentrations of 8% and 10% showed a reduced effect on MB removal compared to the performance before H_2_O_2_ addition. Excessive H_2_O_2_ may increase surface hydration, reducing MB adsorption by competing for active sites on the catalyst. In heterogeneous catalytic systems, H_2_O_2_ can influence the catalyst’s surface properties, altering its adsorption capacity. Moreover, at high H_2_O_2_ concentrations, partial oxidation may occur, leading to the formation of intermediate compounds that could re-adsorb onto the catalyst or adsorbent. This can block active sites and ultimately reduce MB removal efficiency. Excessive amounts of H_2_O_2_ can hinder the process by reducing available active sites and altering the adsorption properties of the catalyst.

For the initial MB concentration of 10 ppm, as shown in Fig. 4 (a), the optimal H_2_O_2_ concentration was 4%. In comparison, untreated peels without H_2_O_2_ exhibited the lowest removal efficiency of 64%. The addition of 4% H_2_O_2_ significantly enhanced MB removal, achieving an 18.27% improvement compared to adsorption. The results further indicate that for 15 ppm MB, the highest removal efficiency was obtained with 6% H_2_O_2_, which performed slightly better than 4%, showing an increase of less than 1% in degradation efficiency from 4 to 6% as seen in Fig. 4 (a). However, for MB concentrations of 5 and 10 ppm, the optimal removal was achieved with 4% H_2_O_2_. This suggests that as MB concentration rises to 15 ppm, 4% H_2_O_2_ becomes insufficient for effectively breaking down the MB molecules, making 6% H_2_O_2_ the most effective concentration. This could be due to MB dye molecules covering manganese active sites on the catalyst at higher concentrations, thereby decreasing catalytic activity.

**Fig. 5:**
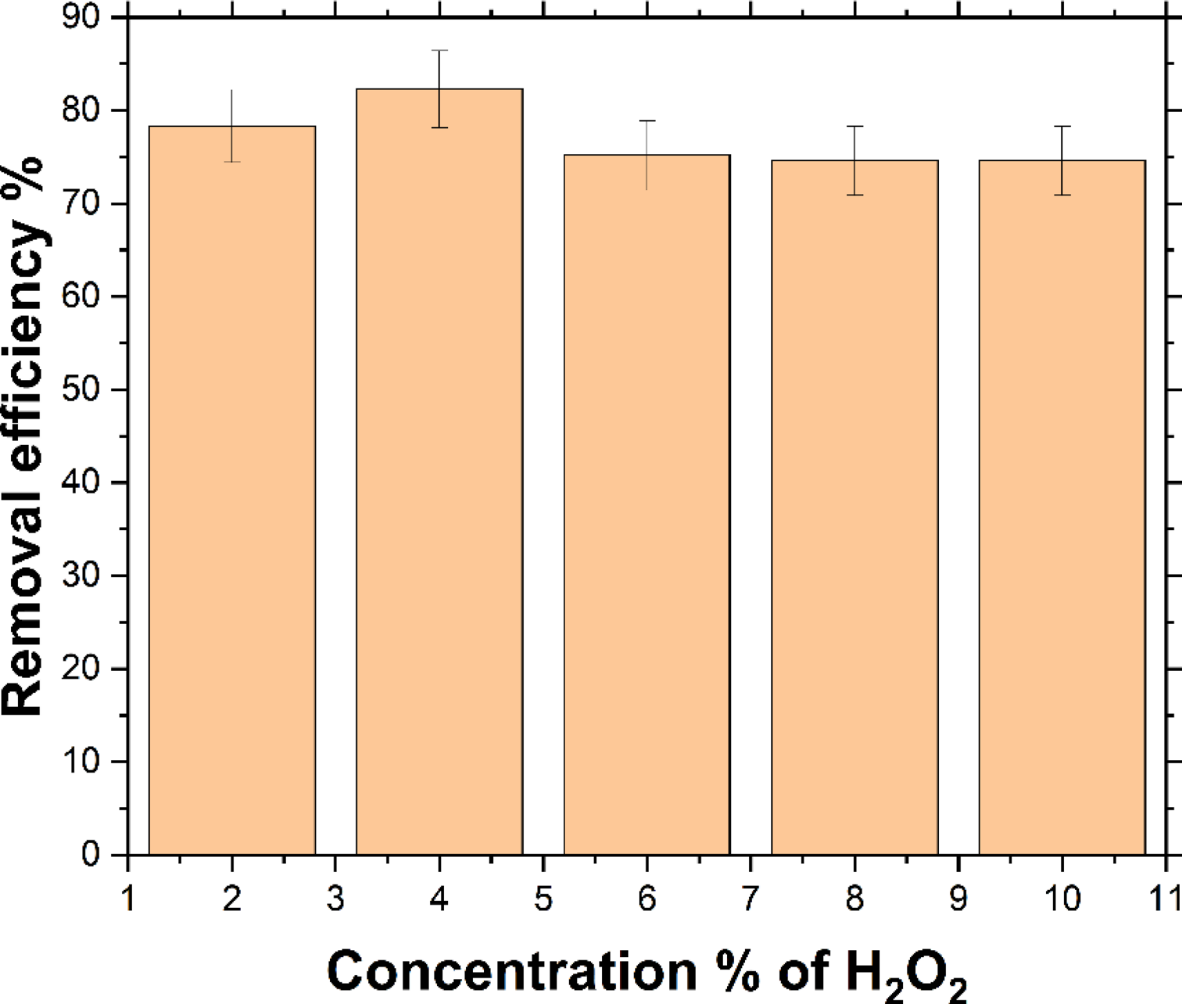
Effect of initial different concentrations of H_2_O_2_ at (2%, 4%, 6%, 8%, and 10%) for (*C*_o_ = 10 ppm at 25 ^o^C).

Therefore, a 4% concentration of H_2_O_2_ yields nearly the same results as 6% at 15 ppm, making it the optimal percentage for all three concentrations at 25 °C. As shown in Fig. 5, with MB concentration of 10 ppm at 25 °C and varying H_2_O_2_ concentrations, the ideal percentage is 4%.

#### Effect of temperature

The temperature variation is a crucial element in determining the design conditions for MB removal in catalytic degradation reactions; therefore, its impact on degradation efficiency should be evaluated. As presented in Table 3, which examines the impact of temperature variations from 25 °C to 45 °C alongside different initial H_2_O_2_ concentrations (2%, 4%, 6%, 8%, and 10%), the highest removal efficiency was observed at 25 °C for the same MB and H_2_O_2_ concentrations.

**Fig. 6:**
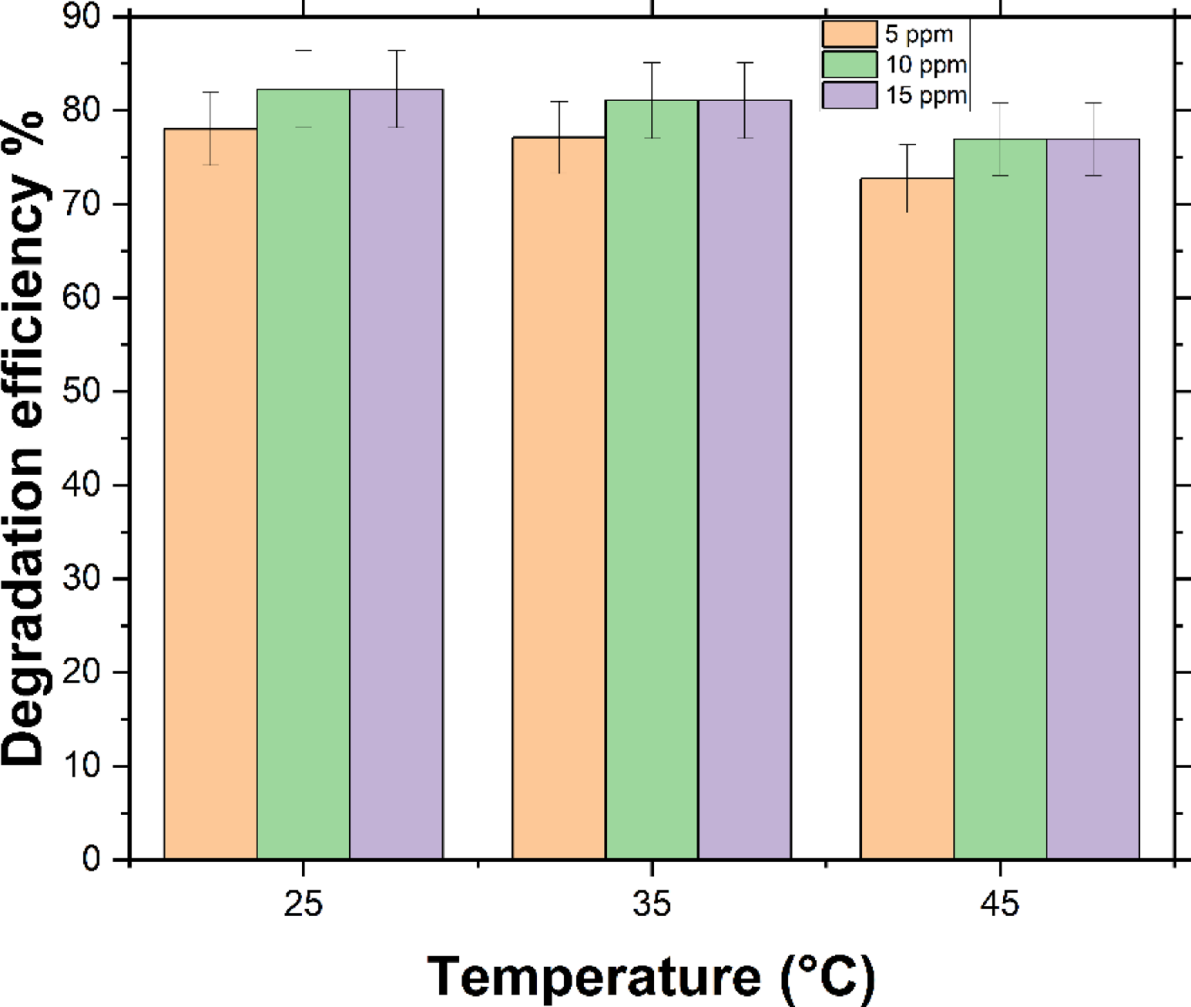
The influence of temperature on MB degradation. (H_2_O_2_ concentration = 4% for *C*_o_ of MB = 5, 10 ppm; and H_2_O_2_ concentration = 6% for *C*_o_ 15 ppm).

Across various H_2_O_2_ concentrations and initial MB concentrations (ranging from 5 to 15 ppm), it was noted that as the temperature increased, degradation efficiency declined. This trend is evident in Fig. 6, for a 6% H_2_O_2_ concentration with 15 ppm MB, as well as for a 4% H_2_O_2_ concentration with initial MB concentrations of 5 and 10 ppm, respectively. The highest removal efficiency was recorded at 25 °C, with a 78.04% removal for 5 ppm MB using 4% H_2_O_2_. Likewise, 82.27% removal was achieved for 10 ppm MB at 25 °C with 4% H_2_O_2_, as detailed in Table 3. For 15 ppm MB, an 83.17% removal was attained with a 6% H_2_O_2_ concentration.

These findings suggest that optimal MB degradation occurs at 25 °C, likely due to the influence of thermal energy. As the temperature rises, the decomposition rate of H_2_O_2_ increases, reducing the availability of reactive hydroxyl radicals essential for breaking down MB molecules, ultimately lowering the overall degradation efficiency.

#### Effect of initial concentration of MB

Due to the fluctuating organic concentration in wastewater, the impact of altering the initial MB concentration was analyzed. As illustrated in Fig. 4, the study considered an initial concentration range of 5–15 ppm. It was observed that as the initial MB concentration increased, the removal efficiency also improved for each H_2_O_2_ concentration (2–10%) at 25 °C, as shown in Fig. 4 (a). Specifically, a higher concentration of 15 ppm enhances the driving force, leading to increased mass transfer and reduced degradation resistance for 2%, 4%, 6%, 8%, and 10% H_2_O_2_ concentrations. This improvement in MB removal can be attributed to the greater availability of active sites on the catalyst, enabling more effective interactions between the organic matter and the oxidizing agents^33,66^.

At 35 °C, as depicted in Fig. 4 (b), the degradation efficiency of MB initially increases with higher MB concentrations, particularly for 5 and 10 ppm. However, when the MB concentration reaches 15 ppm, the efficiency declines at H_2_O_2_ concentrations of 2% and 4%. This reduction may be due to the lower H_2_O_2_ concentrations being insufficient to effectively degrade MB at elevated initial concentrations. Conversely, when the H_2_O_2_ concentration is raised to 6%, the degradation efficiency improves again for the 15 ppm MB concentration.

At 45 °C, as shown in Fig. 4 (c), the degradation efficiency continues to rise with increasing MB concentrations, especially at H_2_O_2_ concentrations of 4% and 6%. However, at a lower H_2_O_2_ concentration of 2%, the efficiency starts to decline once the MB concentration reaches 15 ppm. This trend suggests that, at lower H_2_O_2_ concentrations, sufficient reactive radicals are generated to effectively degrade MB at 5 and 10 ppm, enhancing efficiency. However, at 15 ppm, the available H_2_O_2_ becomes inadequate to sustain radical production, leading to reduced degradation efficiency as reactive species are depleted relative to the higher MB concentration.

Moreover, as the temperature rises to 35 °C and 45 °C, degradation efficiency decreases for the higher MB concentration of 15 ppm when using the lowest H_2_O_2_ concentrations. This decline may be attributed to excessive thermal energy, which accelerates the premature decomposition of H_2_O_2_, thereby reducing the availability of reactive radicals essential for MB degradation. Additionally, elevated temperatures may result in the rapid consumption of radicals, limiting their effectiveness in breaking down higher MB concentrations. Consequently, degradation efficiency diminishes when the reaction conditions exceed the optimal temperature range^67^. Therefore, the impact of temperature should be examined, as it is a crucial factor influencing the degradation efficiency of MB.

**Fig. 7:**
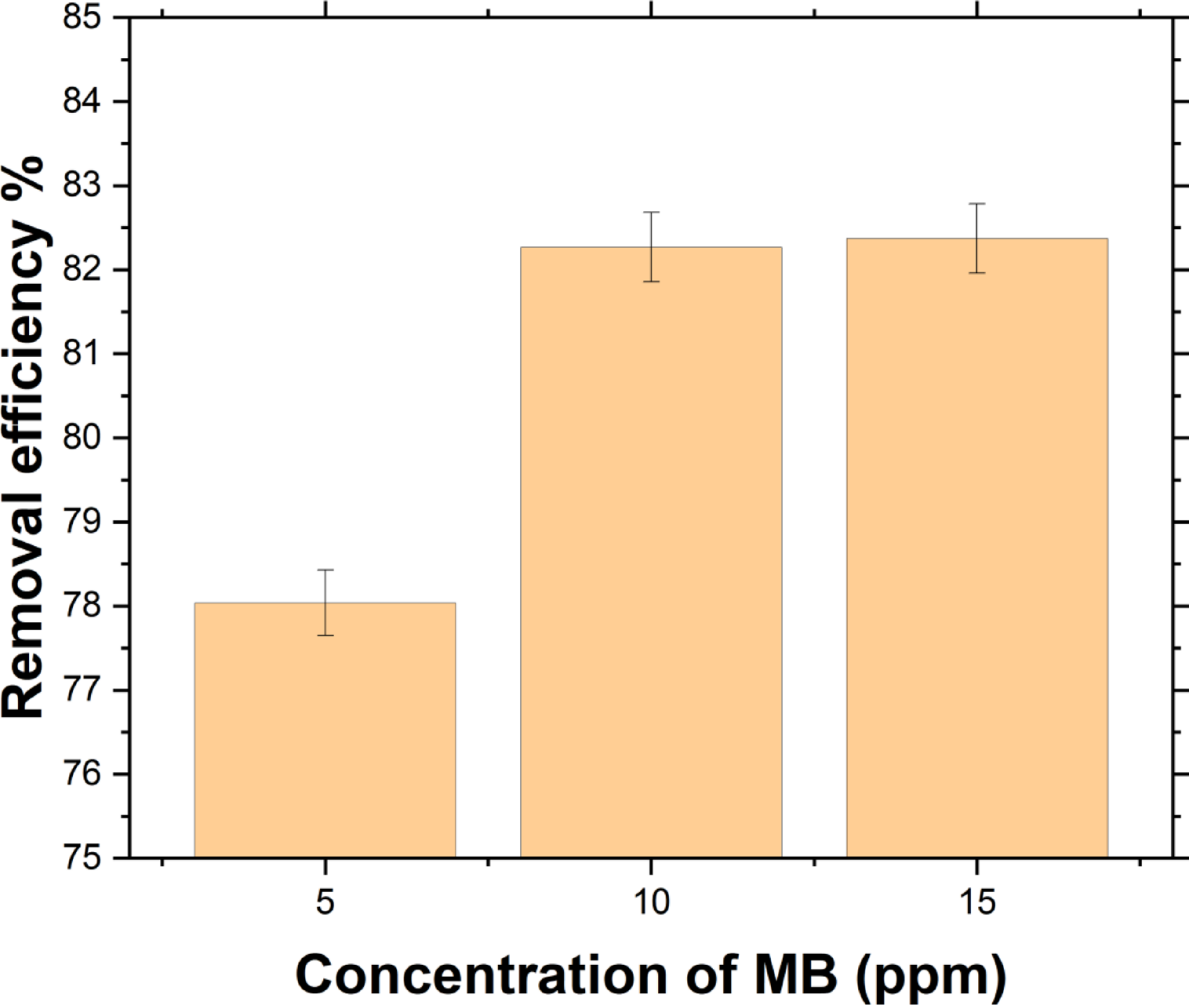
Effect of initial different concentrations of MB (*C*_o_ = 5, 10, and 15 ppm at 25 ^o^C) at 4% H_2_O_2_ and 25 ^o^C.

All in all, the optimal H_2_O_2_ concentration is 4% at 25 °C. As illustrated in Fig. 7, increasing the initial concentration from 5 to 15 ppm improved degradation efficiency, rising from 78.04% at 5 ppm to 82.27% at 10 ppm, followed by a slight increase to 82.37% at 15 ppm.

**Table 3:** Outputs of changing temperature for different concentrations of H_2_O_2_ from 2 − 10% for initial concentration of MB with *C*_o_ ranges from 5–15 ppm.

Removal efficiency %			
Temperature (°C)	5 ppm	10 ppm	15 ppm
Concentration of H_2_O_2_ = 2%			
25	75	78.3	79.44
35	74.65	77.18	76.22
45	69.62	76.06	75.56
Concentration of H_2_O_2_ = 4%			
25	78.04	82.27	82.37
35	77.13	81.08	79.09
45	72.74	76.95	78.99
Concentration of H_2_O_2_ = 6%			
25	73.84	75.17	83.17
35	72.55	74.88	79.84
45	71.54	74.58	77.27
Concentration of H_2_O_2_ = 8%			
25	63.4	74.58	81.86
35	63.13	73.4	74.3
45	63.03	73.04	74.3
Concentration of H_2_O_2_ = 10%			
25	63.4	74.58	81.86
35	63.13	68.67	73.95
45	63.03	68.67	73.55

There is a research study near to our experiment from a conditions point of view which is degradation of MB though nano metallic particles at 25 °C and 10 ppm of MB where the rate of MB degradation with Nano metallic particle (NMP) was very low without added H_2_O_2_, likely due to the absence of hydroxyl radicals in the reaction. MB degradation efficiency increased from 71.6 to 99.6% with rising H_2_O_2_ concentrations from 0 to 100 mM, due to the generation of a significant number of hydroxyl radicals^68,69^. However, the efficiency slightly decreased with 150 mM of hydrogen peroxide. This decline is attributed to the recombination of hydroxyl radicals, the scavenging effect of H_2_O_2_, and the inhibition of NMP oxidation by H_2_O_2_ Eqs. (2–4). At high H_2_O_2_ concentrations, a new radical (HO˙_2_) is formed, which is less reactive than hydroxyl radicals Eq. (3)^37^. Additionally, HO˙_2_ contributes to the destruction of hydroxyl radicals, while two hydroxyl radicals can recombine to form H_2_O_2_ Eq. (2) and Eq. (4)^70–72^.

#### MB degradation by S2

Based on a review of the investigated studies of MB removal experiments for S1, the optimal conditions for S2 were identified. The highest degradation efficiency was achieved at 5 and 10 ppm with a 4% H_2_O_2_ concentration, while for 15 ppm, the maximum removal efficiency occurred with a 6% H_2_O_2_ concentration. All these conditions proved to be most effective at 25 °C regardless of the different concentrations of H_2_O_2_ and MB. These selected conditions will be implemented for S2 to evaluate the effect of a 5% NaOH solution and its potential to enhance the degradation process. Hence, a 4% H_2_O_2_ concentration was applied for 5 and 10 ppm MB, whereas a 6% concentration was applied for 15 ppm MB for S2.

**Fig. 8:**
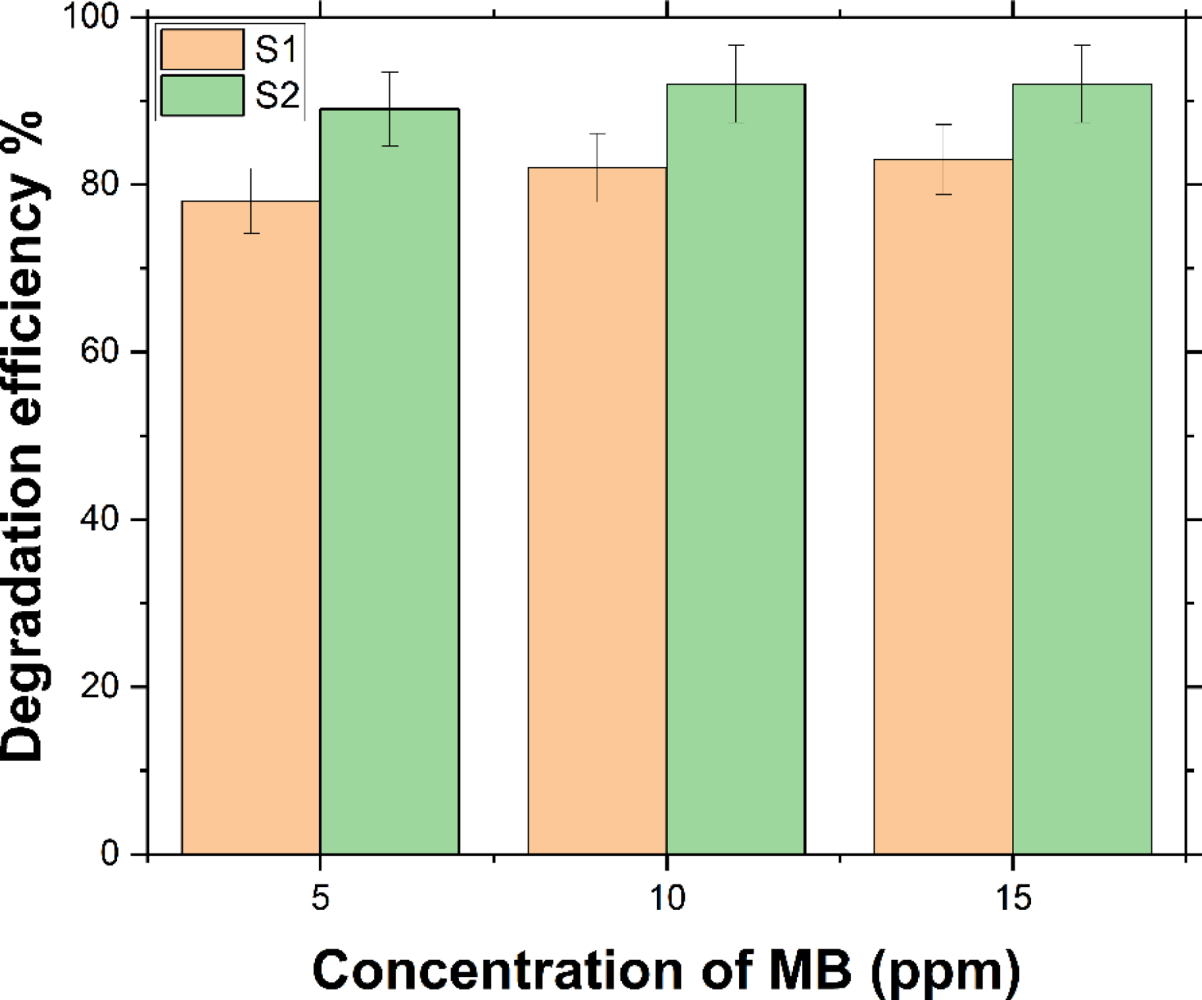
Degradation efficiency of MB of S1 Versus S2 at 25 ^o^C with Concentration of 4% H_2_O_2_ for *C*_o_ = 5 and 10 ppm; and 6% for 15 ppm.

As illustrated in Fig. 8, increasing the initial MB concentration from 5 to 15 ppm led to an improvement in degradation efficiency either for S1 or S2 samples. With 5% NaOH treatment, the MB concentration decreased from 5 to 0.549 ppm, achieving an 89.02% removal efficiency. Under the same conditions (25 °C, 4% H_2_O_2_), S1 achieved a 78.04% removal, reflecting 11% improvement with S2. Similarly, for an initial concentration of 10 ppm, NaOH treatment reduced the MB concentration to 0.804 ppm, resulting in a 91.96% degradation efficiency, whereas S1 exhibited an 82.27% removal, indicating a nearly 10% enhancement. For 15 ppm, the concentration dropped to 1.216 ppm with NaOH treatment at 25 °C and 6% H_2_O_2_, achieving a 91.9% removal efficiency, while S1 showed 83.17% removal, reflecting a 9% improvement. Although the removal efficiency increased with higher MB concentrations, it plateaued at 10 and 15 ppm yielding nearly identical results. The removal efficiency of MB was 90% for 5 ppm and around 92% for both 10 and 15 ppm, as shown in Fig. 8.

The improvement in removal efficiency after 5% NaOH treatment can be attributed to several factors. EDX analysis confirmed that NaOH treatment increased the manganese atomic percentage in lupine peels from 0.1 to 0.2%, enhancing the availability of active sites for H_2_O_2_ decomposition and, consequently improving MB degradation. This alkaline treatment modifies the chemical composition of the peels, increasing active sites and boosting catalytic activity by elevating manganese content. As a result, H_2_O_2_ decomposition into reactive hydroxyl radicals is facilitated, optimizing surface properties for better interaction with H_2_O_2_. This ultimately leads to higher MB removal efficiency compared to S1. Furthermore, XPS analysis indicated that after NaOH treatment, transition metals became more concentrated in the surface layers, enhancing the decomposition of H_2_O_2_. The synergy between surface functionalization (oxygenated and nitrogenous species), trace metal catalysis, and structural stability (due to silica) enhances S2’s ability to degrade MB efficiently. These features improve H_2_O_2_ activation (ROS production) and pollutant adsorption, leading to superior removal performance.

According to the above results, Lupin peels can be considered attractive and effective AOP material. Table 4 gives a comparison with some other different AOP materials whose decomposition mechanism is based on a Fenton-like reaction. Lupin peels material shows a notable time-based performance.

**Table 4:** MB-degradation comparison with other catalysts using.

Reference	Material (source)	Performance	Mechanism	Evaluation (time-based)	Limitation
^73^	iron phosphide (FeP) ***(synthetic*** **)**	Applicable in broad pH range (3.0 − 9.0), capable of degrading 95.74% and 89.01% of MB with 100 ppm within 30 s at pH 3 and 5, and 99.65% and 90.06% within 3 min at pH 7 and 9.	Fenton-like (Fe^III^/Fe^II^ cycles)	Outstanding: 30 s to 3 min	Synthetic FeP
^74^	Natural Zeolite-Based Silver and Magnetite Nanocomposites ***(natural/synthetic)***	Kinetics of MB solutions discoloration by the modified zeolites: experiments take 150 min to study.	Fenton-like	Moderate: 150 min	Partially synthetic
^75^	Zinc oxide (ZnO) ***(synthetic*** **)**	Kinetics of MB solutions discoloration by ZnO: experiments take 150 min to study.	Fenton-like synergism with photocatalytic effect	Moderate: 150 min	Synthetic ZnO
**Present work**	Lupin peels leftover **(bio-natural)**	Kinetics of MB solutions discoloration by Lupin peels: experiments take 100 min to study.	The Fenton-like reaction through manganese (Mn^IV^/Mn^III^ cycles)	Effective: 10–30 min (Fig. 3. (b) and (c))	Probable release of some minerals (however, natural)

#### The proposed mechanism of MB degradation through Mn active sites acting as a Fenton-like catalyst

The novelty of mineral elements especially Mn in lupine peels revealed by XPS, plays a key role in oxidation-reduction reactions, facilitating the generation of reactive hydroxyl radicals for the degradation of MB molecules. Given that manganese has the highest atomic percentage (0.26%) compared to iron and copper, it likely plays a more significant role in the Fenton-like reaction than the other two metals. The oxidative degradation of MB is known to occur as a surface reaction that produces ROS^76,77^.

This process follows a Fenton-type mechanism, where oxidation involves the generation of free radicals from an oxidizing agent and a substance capable of donating electrons^76,78^. The proposed mechanism suggests that acid-base reactions occur on the surface of lupine peels, which contain Mn^4+^ active sites. Initially, an acid-base interaction takes place between one of the oxygen atoms in H_2_O_2_ and Mn^4+^^20,21^. This is followed by electrostatic attraction between the hydrogen atoms of H_2_O_2_ and the negatively charged layered material, enhancing the decomposition of H_2_O_2_ into reactive species.

These findings indicate that MB degradation is primarily driven by the production of (ROS) from H_2_O_2_, particularly on MnO_2_ in lupine peels, generating singlet oxygen (^1^O_2_)^12,34^ and free radicals like ˙OH and HOO˙^20,21,78^. The formation of ˙OH radicals further accelerates the breakdown of MB, leading to its complete mineralization^12,79,80^. The expected mechanism of the Fenton-like reaction through manganese is outlined in the following Eqs. (5) to (10)^81–83^.

5 $${\text{Mn}}^{{{\text{4}} + }} + {\text{H}}_{{\text{2}}} {\text{O}}_{{\text{2}}} \to {\text{H}}^{ + } + {\text{ HOO}^\cdot} + {\text{Mn}}^{{{\text{3}} + }}$$

6 $${\text{H}}_{{\text{2}}} {\text{O}}_{{\text{2}}} + {\text{ HOO}^\cdot} \to {^\cdot\text{OH}} + {\text{H}}_{{\text{2}}} {\text{O }} + {\text{ O}}_{{\text{2}}}$$

7 $${\text{HOO}^\cdot} \rightleftharpoons {\text{H}}^{ + } + {\text{ O}}_{{\text{2}}} ^{{-}}$$

8 $${\text{H}}_{{\text{2}}} {\text{O}}_{{\text{2}}} + {\text{ O}}_{{\text{2}}} ^{{-}} \to {\text{ OH}}^{{-}} + {\text{ OH }} + {\text{O}}_{{\text{2}}}$$

9 $${\text{Mn}}^{{{\text{3}} + }} + {^\cdot\text{OH }} \to {\text{Mn}}^{{{\text{4}} + }} + {\text{OH}}^{{-}}$$

10 $${\text{MB}}^{ + } + {^\cdot\text{OH}} \to {\text{ H}}_{{\text{2}}} {\text{O }} + {\text{ CO}}_{{\text{2}}} + {\text{ oxidized products}}$$

**Fig. 9:**
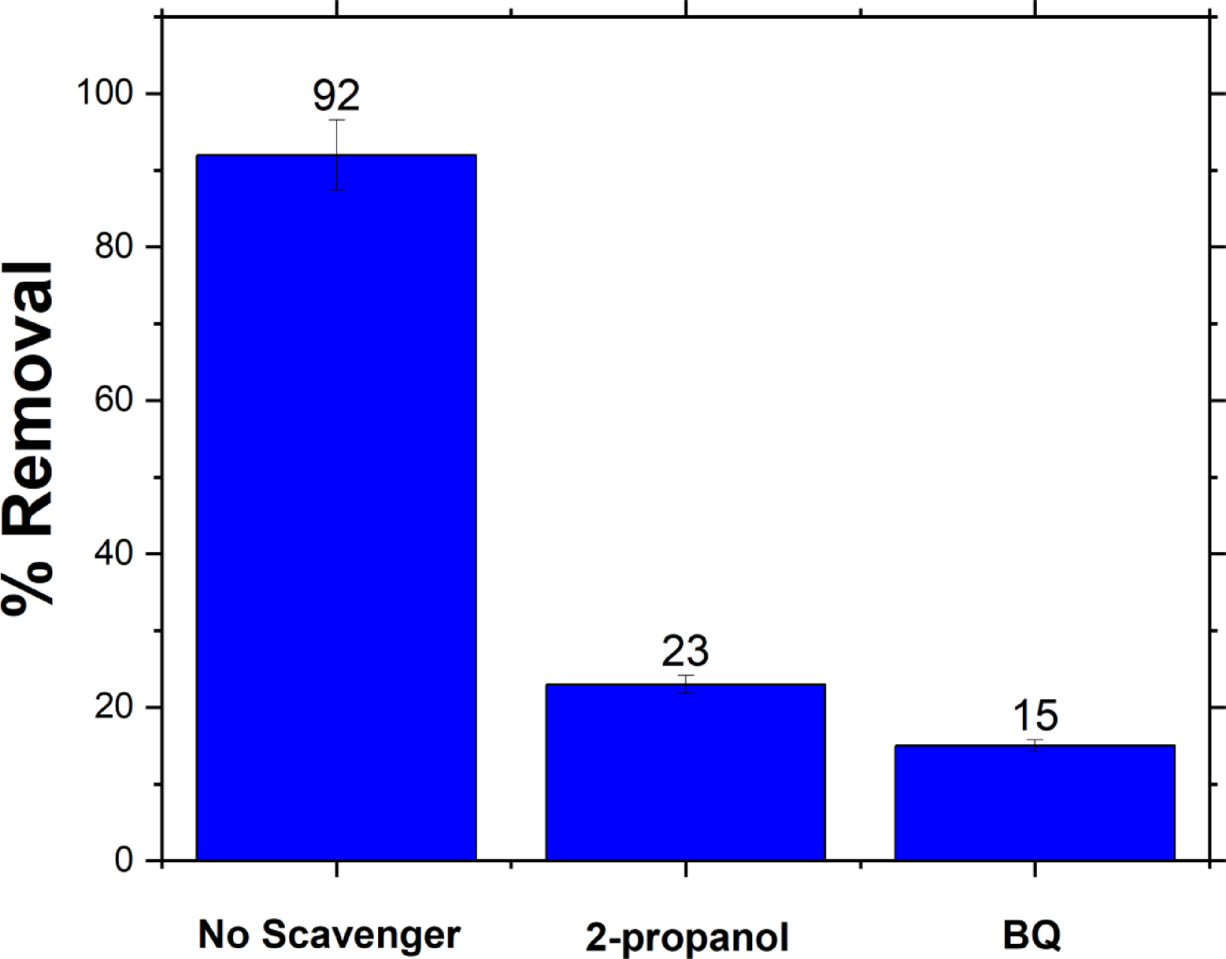
The scavenging experiment of the active species during catalytic degradation of MB (5 ppm) with the addition 2- propanol as scavengers for ·OH, and 1,4-benzoquinone (BQ) as scavengers for HO₂· radicals.

In general, ·OH and HO₂· radicals are recognized as the primary reactive species in catalytic oxidation processes. The hydroxyl radical (·OH) is a potent oxidizing agent for a wide range of organic compounds. In this study, 2-propanol and 1,4-benzoquinone (BQ) were employed as scavengers for ·OH and HO₂· radicals, respectively^84^. These scavengers were added to the reaction system to trap the respective radicals during the degradation of MB. As shown in Fig. 9, the presence of these scavengers led to a significant reduction in MB degradation efficiency from 92% down to 23% with 2-propanol and 15% with BQ. These findings indicate that the degradation of MB is strongly reliant on the presence of ·OH and HO₂· radicals.

## Conclusion

The lupine peel catalyst was investigated as a novel for its catalytic activity, as it contains transition metals like manganese, identified through XPS characterization. These metals serve as active sites for H_2_O_2_ decomposition, leading to the formation of ROS that facilitate MB degradation. Several factors influence the removal efficiency of MB, including temperature, H_2_O_2_ concentration, and the initial MB concentration. To determine the optimal conditions for MB decolorization, these parameters were systematically studied, considering that MB concentration varies in wastewater. Therefore, the selection of optimal temperature and H_2_O_2_ concentration depends on wastewater conditions. The best conditions identified for S1 were applied to S2, as XPS analysis revealed that S2 has a higher concentration of transition metals hence, enhancement of reactive sites on the peel surface which accelerates H_2_O_2_ decomposition. Additionally, S2 enhances MB removal efficiency due to its eroding effect, which alters the elemental composition, functional groups, and surface morphology. Limited studies have explored the use of agricultural waste as a standalone natural catalyst for degradation processes. Typically, such materials are either impregnated with transition metals or utilized for their inherent peroxidase content. This suggests a promising avenue for future research, emphasizing the characterization and experimental validation of agricultural waste as a naturally reusable catalytic material.

## Data Availability

The datasets used and/or analyzed during the current study are available from the corresponding author Osama Abuzalat on reasonable request.
